# Case of Stomatitis Materna and Intricate Diarrhœa Complicating Each Gestation and Lactation; with Dropsy of the Amnion, and Premature Expulsion of an Emphysematous Living Fœtus in the Last Pregnancy

**Published:** 1876-12

**Authors:** J. Schneck

**Affiliations:** Mt. Carmel, Ill.


### HISTORY OF A CASE OF STOMATITIS MATERNA AND INTRACTABLE DIARRHŒA COMPLICATING EACH GESTATION AND LACTATION; WITH DROPSY OF THE AMNION, AND PREMATURE EXPULSION OF AN EMPHYSEMATOUS LIVING FŒTUS IN THE LAST PREGNANCY.

By J. SCHNECK, M.D., of Mt. Carmel, Ill.

(Read before the Union Medical Society, June 27, 1876.)

Case: Mrs. M. S., aged 28, medium size, blonde, unusually fair skin, was confined with her first child, March 10, 1871. Soon after she discovered a sudden appearance of small blisters on the tongue and mucous lining of the mouth and cheeks; in a few hours they would discharge their contents, leaving an abraded fissured surface of a deep red color, very sensitive to the blandest articles of ingesta; even water or cold air would cause a feeling as if "red hot."

From that time to this, there has never been a time when the mouth could be said to be entirely free from this unnatural tenderness; being worse while nursing, and during the last four or five months of each succeeding pregnancy.

In a few days after the stomatitis had commenced, she was attacked with a diarrhœa, with at first semifluid stools and colicky pains with excessive borborygmus. Soon she had frequent passages consisting of mucus and half digested food, accompanied with severe griping, tenderness of abdomen, and neuralgic (probably partially hysteric) pains in different parts of the body, which were only relieved by large doses of opium.

The patient, in spite of treatment, gradually grew weaker and worse, until the end of the fifth month after confinement. On the death of her babe, Aug. 15, 1871, she commenced improving and in a few months had regained her former good health, except the tenderness of the mouth.

About Aug. 10, 1872, the woman again being pregnant, the mouth become worse, the intractable diarrhœa returned and soon assumed its former virulence; no treatment appeared of any permanent value; large doses of opium and other narcotics were necessary to give temporary relief. On Dec. 15, 1872, she was delivered of a healthy child, apparently at full term. The diarrhœa, however, continued, the patient growing weaker and more emaciated.

March 8, 1873, I saw the patient for the first time, and found her very weak, unable to raise herself in bed, with a quick and weak pulse, fever during the day-time and night sweats. There was constant, burning sensation at the stomach, the bowels were swollen and tender, and when not under the influence of narcotics, excessively painful. The bowels moved from four to twelve times daily, the stools consisting mostly of bloody mucus and undigested food. The child was in ill health, very much emaciated, and nursed by its mother.

The child was ordered to be weaned, and the mother to have before each meal a powder of pepsin, bismuth subnitrate and prepared chalk, of each two grains. She was also to have regularly, elixir of iron, quinia and strychnia, and cod-liver oil. At the end of five weeks of treatment the patient had so far convalesced as to be able to perform her household duties, and soon recovered her usual health, in which she continued until her next gestation — nearly two years.

She menstruated last during the last week of October, 1875, suffered moderately from nausea and morning sickness during November and December, otherwise felt moderately well until March, 1876, when the same difficulty as in former pregnancies commenced. This time the stomatitis was not so severe as in the two previous gestations, while the enteric symptoms were worse.

She felt motion first during the first week of April. I saw her April 18, and found the mucous membrane of the mouth very red and painful, with deep fissures at the edge of the tongue; frequent dysenteric stools and great weakness. I ordered cod-liver oil three times a day, and one desert spoonful of the syrup of the hypophosphites (Churchill's) after each meal; also an emulsion of turpentine with opium and carbolic acid, a dose to be taken after each motion of the bowels.

On April 30th I found all the symptoms worse, the patient rapidly failing. The abdomen was as large as at full term.

The cod-liver oil was omitted in the treatment. I suggested the advisability of bringing on premature labor, as the only hope of saving her life.

On May 10th at my suggestion, Dr. J. J. Lescher was called in consultation. He believed the only reasonable hope of saving her life was in premature labor; but suggested a trial of powders of opium, 1 gr., and oxalate of cereum, 2 grs., three times daily in addition to the other treatment.

May 12th, I found the patient suffering from severe pains in the bowels and back, great dyspnœa, bowels not moving as frequently as before the last powders were taken. She drank much water, but passed less than the normal quantity; this had been the case for the last four weeks. The abdomen was much larger and she was unable to sit up on account of its size; at the last examination the skin being perfectly tense. The lower portion of the chest, including part of the sternum and ribs were all greatly involved in the enlargment. Percussion gave a dull sound from sternum to pubes, and from the right to the left hypocondriac region. Auscultation revealed nothing but an occasional striking sound, occasioned by the child striking against the inner surface of the uterus. There was no appearance of anasarca.

The patient positively refused to have premature labor brought on. In addition to previous treatment, diuretics and anodynes were directed.

May 16th, I was called again, and as I entered the door the membranes ruptured, and there was a great gush of water. On examination I found the child born, except the head, which was still in the vagina. When this was removed, there was another discharge of an enormous quantity of water. The placenta which was of unusually large size and very soft, soon followed. The cord was about twice the usual thickness, and very easily torn. The womb contracted well, the abdomen in due time assumed its normal size, showing that the enormous distention was occasioned by the contents of the uterus.

My attention was next directed to the child, which up to this time I had considered as being still-born, and in a state of decomposition. The skin was of a dark, venous color and distended in every part to its utmost limit. Eyes were swollen shut; cheeks encroaching on the nose until it was scarcely recognizable; limbs apparently twice the normal size, it being scarcely possible to bend fingers, arms or legs without breaking the skin. Pressure gave a feeling as if pressing on a half-solid India rubber ball, making an indentation on the surface, it would suddenly spring back to its former shape, showing that the distention was not caused by serum. While examining the child it made three separate and distinct efforts at breathing, to the utter astonishment of all present; but respiration was not established. The mother rapidly recovered her former health, except that the tenderness of the mouth still continues.

The above case is of unusual interest for the following reasons:

1. As pointing to a common cause of the stomatitis materna and the intractable form of diarrhœa that sometimes occur during gestation and lactation. The cause of neither so far as I am aware, has ever been satisfactorily explained.

2. As adding another, to the small number of reported cases of dropsy of the amuion. The cause of this has also called forth many ingenious theories, but none so far have been satisfactory to the profession.

3. But especial interest is attached to the fact of a *live born emphysematous fœtus.* I have examined eight[1] of our most prominent authors on midwifery and in none of them have I found any reference to a similar case. Leishman, Ramsbotham and Cazeaux, all speak of a large accumulation of gas in the abdominal and thoracic cavities of the fœtus as sometimes obstructing labor. And Depaul is quoted by the last named author as having had a case where there was general emphysema involving all the cellular tisue; but in every instance they speak of it as the result of death and commencing decomposition.

[1] Schroeder, Manual of Midwifery, 1873; Churchill's (by Condie) System of Midwifery, 1851; Ramsbotham, Process of Parturition, 1842; Lee, Theory and Practice of Midwifery, 1844; Meigs, Treatise on Obstetrics, 1856; Moreau, Practice of Midwifery, 1844; Cazeaux, Theoretical and Practical Midwifery, 1868; Leishman, System of Midwifery, 1873.

That there were three separate and distinct efforts to breathe after its birth, by the fœtus in this case, there cannot be a shadow of doubt, there having been three persons beside myself who witnessed it. Neither do I think that the phenomenon above referred to can be explained on the ground of the muscular irritability which sometimes continues for a short time after death, as this is known to be totally lost when decomposition commences, and before gas is evolved.

[We regret that on the question as to whether the fœtus was alive at birth, the author has not told us whether its heart was beating, also that he did not puncture the skin immediately after death, and thereby establish beyond a peradventure the nature of the material distending the cellular tissue.—Eds.]

